# Mechanisms of action for different checkpoint inhibitors

**DOI:** 10.1097/HS9.0000000000000244

**Published:** 2019-06-30

**Authors:** Pedro Berraondo

**Affiliations:** 1 Program of Immunology and Immunotherapy, Cima Universidad de Navarra, Pamplona, Spain; 2 Navarra Institute for Health Research (IDISNA), Pamplona, Spain; 3 Centro de Investigación Biomédica en Red de Cáncer (CIBERONC), Spain

**Take home messages**

- Monoclonal antibodies targeting CTLA-4 enhance the activation of T lymphocytes. The toxicity of these monoclonal antibodies can be reduced targeting the antibody activity to the tumor microenvironment.
- Monoclonal antibodies targeting the PD-1/PD-L1 axis normalize the effector immune responses in the tumor microenvironment. These monoclonal antibodies are essential drugs for therapeutic combinations due to the excellent safety and efficacy profile of the PD-1/PD-L1 blockade.

## Introduction

The immune system is composed of a complex network of cells and soluble factors. This network is specialized in detecting dangerous homeostatic alterations. Once a dangerous alteration is detected, the immune network initiates an immune response to re-establish the homeostasis.^1▪^ Co-inhibitory receptors control the activation and the intensity of the adaptive immune response and therefore, they function as immune checkpoints. The activity of the immune checkpoints is crucial to avoid exacerbated immune responses and autoimmunity by induction of T lymphocyte exhaustion. This process is mainly induced by the chronic exposure to the antigen and is characterized by (i) the progressive loss of the production of proinflammatory cytokines such as tumor necrosis factor alpha and interferon gamma, (ii) the loss of the cytotoxic activity, (iii) the decrease in the proliferative potential and (iv) an increase in apoptosis. Exhaustion is a progressive process that can finally lead to the clonal deletion of T lymphocytes with high-affinity T cell receptor (TCR). The T lymphocytes express progressively co-inhibitory receptors such as PD-1, LAG-3, and TIM-3. This process also involves an epigenetic remodeling that marks a point of terminal exhaustion.^2^

**Figure 1:**
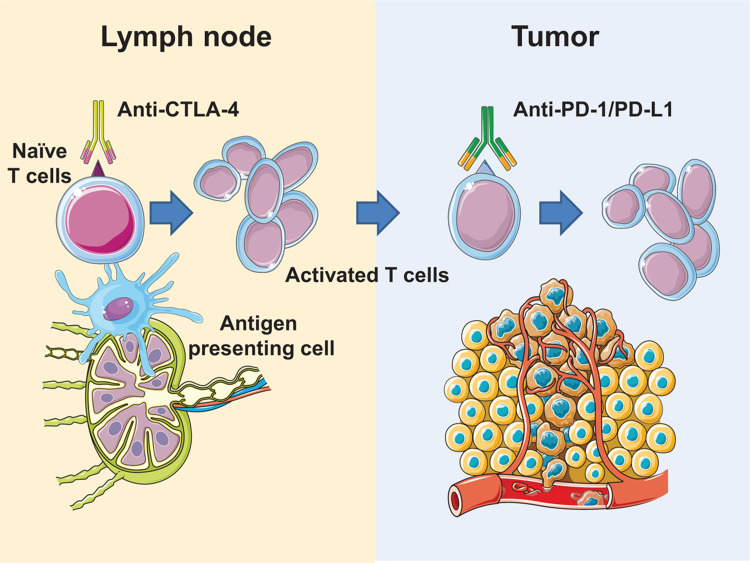
**Mechanisms of action for CTLA-4 and PD-1**. (A) CTLA-4 acts at the priming phase of the immune response enhancing the activation of T lymphocytes. (B) The main anatomical site of PD-1 activity is the tumor microenvironment. Monoclonal antibodies targeting PD-1/PD-L1 normalize the antitumor immune response. This figure contains elements from Servier Medical Art.

Monoclonal antibodies (mAbs) targeting 2 of these immune checkpoints (cytotoxic T lymphocyte antigen 4 (CTLA-4) and programmed cell death protein 1 (PD-1)) have been approved for the treatment of several malignancies. The clinical activity of these monoclonal antibodies is characterized by an excellent safety profile and by the induction of durable response in a fraction of patients. Due to the relevance of these clinical results, James Allison and Tasuku Honjo were awarded the Nobel prize in Medicine in 2018 for the discovery of CTLA-4 and PD-1 (Fig. 1).

## Current state of the art

CTLA-4 acts at the priming phase of the activation of naïve T lymphocyte and is also a fundamental mediator of the suppressor activity of regulatory T lymphocytes. It is a co-inhibitory molecule of great relevance since murine models deficient in CTLA-4 develop lethal autoimmune processes characterized by T cell activation and infiltration in multiple organs.

CTLA-4 belongs to the immunoglobulin superfamily and is expressed mainly in T cells. In naïve T lymphocytes, CTLA-4 is localized in intracellular vesicles. Activation of T lymphocytes through TCR signaling induces CTLA-4 translocation to the plasma membrane via exocytosis. This process is dependent on the intensity of the activatory signal. In regulatory T lymphocytes, CTLA-4 is expressed constitutively and is essential for its immunosuppressive functions and maintenance of peripheral tolerance.

CTLA-4 is homologous to CD28 with higher affinity for CD80 or CD86. Unlike CD28, CTLA-4 does not transduce a co-stimulatory signal. On the contrary, it acts as a competitive inhibitor that prevents CD28-mediated signaling, lowers CD28 levels in the antigen-presenting cell, or initiates an inhibitory signaling pathway. Furthermore, it has been described that CTLA-4 can increase the mobility of the T lymphocyte during the immunological synapse, thereby destabilizing the interaction with the antigen-presenting cell. As a consequence of all these mechanisms of action, there is a decrease in the production of IL-2, proliferation, and survival of the T lymphocyte. Therefore, the balance of the interaction between CD28:CD80/CD86 vs CTLA4:CD80/CD86 during the immunological synapse determines if the lymphocyte is activated or becomes anergic.^3▪^

PD-1 plays a crucial role in peripheral tolerance protecting the organism from exacerbated immune responses and autoimmunity. Alterations in the PD-1 signaling pathway have a great impact on immunological homeostasis. Murine models deficient in PD-1 develop accelerated autoimmunity. In contrast, sustained expression of PD-1 and its ligands are common in chronic viral infections and cancer. mAbs that block the PD-1/PD-L1 axis enhance the T lymphocyte function, decreasing viral load and tumor size. Unlike CTLA-4, PD-1 regulates the function of previously activated T lymphocytes and mainly in peripheral tissues. PD-1 is expressed in all populations of T-lymphocytes during their activation, in B-lymphocytes, NK cells and some types of myeloid cells. The intensity of expression of PD-1 increases progressively during the exhaustion of T lymphocytes.

The PD-1 ligands known as PD-L1 and PD-L2 are expressed both in hematopoietic cells (dendritic cells, macrophages, T cells, and B cells) and in non-hematopoietic cells (endothelial cells, keratinocytes, pancreatic islets, and cancer cells). Proinflammatory signals induce the expression of the PD-1 ligands, being type I and II interferons the main inducers. In addition to the induction of expression through this adaptive mechanism of resistance, ligands can be intrinsically expressed due to genetic rearrangements in the tumor cell. This is the case of Hodgkin lymphoma, in which due to amplifications on chromosome 9p24, ligands PD-L1 and PD-L2 are constitutively overexpressed.

The cytoplasmic tail of PD-1 contains 2 structural motifs: ITIM and ITSM. Once PD-1 interacts with its ligands, the tyrosine residues are phosphorylated, which allows the recruitment of cytoplasmic tyrosine phosphatases such as SHP2. These phosphatases antagonize the signal of the TCR and CD28, altering the signaling of the PI3K, ERK, RAS, VAV, and PLCγ pathways. As a consequence, there is a decrease in the activation of the transcription factors AP-1, NFAT, and NF-kB; reducing the activation, proliferation, survival, and production of cytokines in the T lymphocyte.^4▪^ mAbs that block PD-1 activity induce a cytokine cross-talk between the PD-1^+^ T cells and the dendritic cells specialized in cross-priming in the tumor microenvironment.^5^ As a result of the release of proinflammatory cytokines, intratumoral Tcf1^+^ T cells with stem-like properties are expanded.^6^

## Future perspectives

mAbs targeting the PD-1/PD-L1 axis have been approved for multiple solid tumors and hematological malignancies. The exceptional safety profile and antitumor activity in some indications are a milestone in oncology.^7▪^ More than 2000 clinical trials are evaluating the safety and efficacy of anti-PD-1 or anti-PD-L1 monoclonal antibodies with other drugs.^8^ The different therapeutic strategies that are being combined with PD-1 blockade include surgery, radiotherapy, targeted therapies, virotherapy, and other immunotherapies. As a result of this intense clinical research, the combination of anti-PD-1 and anti-CTLA-4 was approved for the treatment of melanoma, renal cancer, and a subgroup of metastatic colorectal cancer.

Anti-CTLA-4 mAb in monotherapy is only indicated for the treatment of melanoma due to a more toxic profile. New strategies to reduce the toxicity of CTLA-4 antibodies will expand the beneficial effects of these mAbs to other indications. Two strategies are being tested in clinical trials. First, the depletion of regulatory T cells in the tumor microenvironment can be achieved by the optimization of the Fc domain of anti-CTLA-4 mAbs to enhance the antibody-dependent cellular cytotoxicity.^9▪^ The second strategy is based on the release of the active moiety in the tumor microenvironment through the use of peptide linkers that are digested by metalloproteinases in the tumor microenvironment.^10^
